# Multimodal assessment of acute stress dynamics using an aversive video paradigm (AVP)

**DOI:** 10.1016/j.ijchp.2025.100607

**Published:** 2025-07-03

**Authors:** Sumit Roy, Yan Fan, Mohsen Mosayebi-Samani, Maren Claus, Nilay Mutlu, Thomas Kleinsorge, Michael A. Nitsche

**Affiliations:** a Department of Psychology and Neurosciences, Leibniz Research Centre for Working Environment and Human Factors, Dortmund, Germany; b International Graduate School of Neuroscience (IGSN), Ruhr University Bochum, Bochum, Germany; c Department of Immunology, Leibniz Research Centre for Working Environment and Human Factors, Dortmund, Germany; d Bioengineering Department, Yildiz Technical University, Istanbul, Turkey; e German Centre for Mental Health, Germany; f Bielefeld University, University Hospital OWL, Protestant Hospital of Bethel Foundation, University Clinic of Psychiatry and Psychotherapy and University Clinic of Child and Adolescent Psychiatry and Psychotherapy, Germany

**Keywords:** Stress, Stress induction paradigm, Aversive video clips, Heart rate variability, Cortisol, Cytokines, EEG

## Abstract

This study explored the efficacy of inducing stress through aversive video clips and investigated its impact on psychological processes, brain, and vegetative physiology. This study had a randomized, single-blinded, crossover design, where 78 right-handed male participants were exposed to aversive or neutral video clips in separate sessions. Subjective feelings of stress were assessed via questionnaires. Electroencephalography (EEG) with 62 electrodes was recorded continuously. EEG power and connectivity changes based on coherence were analyzed. Heart rate (HR) and heart rate variability (HRV) data were obtained during the whole experiment, and saliva was collected for cortisol and cytokine analysis at different time intervals. Subjective data showed increased anxiety and negative affect induced by the aversive video clips, accompanied by elevated salivary cortisol levels after exposure to the stressful clips and decreased HRV. Cytokine levels, however, increased over time in both control and stress conditions, which argues against a stress-specific alteration of cytokines in this specific stress protocol. EEG alterations during stress induction suggest a possible disruption of top-down control and increased bottom-up processing, in line with previous literature. These results suggest that the aversive video paradigm (AVP) is a reliable technique to induce psychological stress in a controlled experimental setting and is associated with stress-specific emotional and physiological changes.

## Introduction

The issue of stress has gained increasing prominence in modern society, as it exerts profound effects on both, physical and mental well-being, and consequently, on society (Candeias et al., 2024; Segerstrom & O’Connor, 2012)**.** These effects extend beyond the realm of cognition, and emotion, extending their implications to physiology and metabolism of the human body (Sapolsky, 2004). Psychological stress can be defined as a state where the individual is challenged by a potential loss of control due to external and internal triggers and experiences an increase in allostatic load (McEwen & Gianaros, 2011). Psychological stressors enhance the activity of the hypothalamic-pituitary-adrenal (HPA) axis and the sympathetic nervous system (SNS) (Russell & Lightman, 2019). Increased activity of the SNS and HPA axis leads to physiological changes in multiple organs, including the cardiac system (Levine, 2022)**,** endocrine secretion (Russell & Lightman, 2019), brain oscillations and activation (Hermans et al., 2014; Katmah et al., 2021), the immune system (Alotiby, 2024), and cognition and behavior (Lupien et al., 2007). Thus, stress exerts its impact on diverse systems and warrants a detailed study of these systems.

Stress is a highly dynamic process, particularly concerning acute stressors (Hermans et al., 2014). Hence, investigating stress dynamics in multiple modalities promises to provide a comprehensive understanding of how various systems interact synergistically to produce stress effects.

Reliable methods for stress induction are essential for studying stress in a controlled laboratory setting. Numerous established methods, such as the Trier Social Stressor task (TSST), Montreal Imaging Stressor Task (MIST), and ScanSTRESS, have been used to induce stress, typically involving social evaluative threats (Berretz et al., 2021; Noack et al., 2019). However, these paradigms often demand extensive training, require a relevant amount of research staff members, and it is not trivial in each case to introduce adequate control conditions. To mimic constant stress exposure with uncontrollable and aversive stimuli, which are relevant in modern society, we sought to focus on more selective *psychological* stress induction by showing aversive movie clips. In this paradigm, participants are exposed to threatening stimuli through aversive videos, without the need for active engagement or social evaluation (Noack et al., 2019; Qin et al., 2009). Such stress paradigms align with the contemporary stress landscape and resonate with Mason's determinants of the human stress response (Mason, 1968)**.** Our choice of the AVP as a stress induction method also originates from the need for an experimental protocol that does not involve cognitive engagement, as this project was part of a larger investigation into the influence of stress on working memory and the potential impact of Non-Invasive Brain Stimulation (NIBS) on these effects.

The AVP has previously been applied to elicit stress responses in human participants due to the novelty, unpredictability, and uncontrollability of the depicted scenes (Gärtner et al., 2014; Noack et al., 2019; Qin et al., 2009). The clip content of the present study closely resembled that used in previous research (Gärtner et al., 2014; Qin et al., 2012). This paradigm is easy to administer and does not require the involvement of more than one experimenter. Moreover, an adequate control condition can be established relatively easily by introducing emotionally neutral video clips with otherwise comparable content. Limited research has explored the impact of this stress induction paradigm on brain dynamics using EEG and its specific stress induction-dependent component. Therefore, our study involved a comprehensive analysis, incorporating subjective, physiological, and immunological data collected during exposure to the AVP.

We aimed to document stress-related psychological and physiological alterations induced by this paradigm in line with previous studies. We expected an increase in subjective anxiety as measured by the State-Trait Anxiety Inventory-State (STAI-S), negative emotions as measured by Positive and Negative affect schedule (PANAS), and saliva cortisol levels (Gärtner et al., 2014; Qin et al., 2009). We furthermore expected decreased HRV and a deceleration of the HR, as reported in previous studies with affective movie stimuli (Bos et al., 2013; Codispoti et al., 2008). We expected moreover a stress-related increase of immune activation concerning saliva cytokine levels (Slavish et al., 2015). We furthermore expected to observe specific EEG alterations. We hypothesized that stress disrupts cognitive top-down control and increases bottom-up processing (De Kloet et al., 2019; Joyce et al., 2025; McRae et al., 2012), and thus expected respective changes of brain frequency power and connectivity, including a reduction of low frequency oscillations, which are known to be involved in top-down control,(Xiong et al., 2023) and increased high frequency oscillations, which are involved in bottom-up processing (Riddle et al., 2019). By analyzing all clips individually, we also aimed to observe the dynamics of these effects. By analyzing resting state (RS) before and after clip presentation, we aimed to elucidate after effects of stress clips, where we expected to observe increased arousal, and a state of increased anxiety after exposure to the stress-inducing intervention (Knyazev et al., 2005).

## Results

### Subjective data

The analysis of variance (ANOVA) conducted for the positive affect scores of the PANAS showed a significant main effect of emotional condition [F (1,77) = 19.29, ***p*** **<** **0.001**, η^2^_p_ = 0.2], a significant main effect of time [F(2.19,168.98) = 55.92, ***p*** **<** **0.001**, η^2^_p_ = 0.42], and significant emotional condition x time interaction effect [F (2.49, 192.14) = 4.606, ***p*** **=** **0.007**, η^2^_p_ = 0.056]. The post-hoc comparisons (shown in Fig. 2a.) revealed a decrease in positive affect ratings in both conditions as compared to the first time-point (baseline), with a greater decrease in the stress condition. Post-hoc tests also revealed a decrease in positive affect scores in the stress condition after presentation of the movie clips as compared to the control. The ANOVA conducted for the negative affect scores of the PANAS showed significant main effects of emotional condition [F (1, 77) = 109.13, ***p*** **<** **0.001**, η^2^_p_ = 0.586], time [F (2.44, 187.64) = 65.62, ***p*** **<** **0.001**, η^2^_p_ = 0.46], and a significant emotional condition x time interaction [F (2.61, 201.24) = 86.61, ***p*** **<** **0.001**, η^2^_p_ = 0.53]. The post-hoc comparisons (shown in Fig. 2b) revealed an increase in negative affect ratings in the stress condition and a decrease in the control condition as compared to the first time-point (baseline). Post-hoc tests also revealed a significant increase in negative affect scores in the stress condition after presentation of the movie clips as compared to the control condition. The ANOVA conducted for the state anxiety scores of the STAI-S showed significant main effects of emotional condition [F (1, 77) = 128.05, ***p*** **<** **0.001**, η^2^_p_ = 0.624], time [F (2.53, 194.57) = 75.625, ***p*** **<** **0.001**, η^2^_p_ = 0.495], and a significant emotional condition x time interaction [F (2.55, 196.27) = 95.09, ***p*** **<** **0.001**, η^2^_p_ = 0.533]. The post-hoc comparisons (shown in Fig. 2c.) revealed an increase in anxiety scores in the stress condition and no change in the control condition as compared to the first time-point (baseline). Post-hoc tests also revealed a significant increase in anxiety scores in the stress condition after the movie clips, as compared to the control. Subjective scores thus showed a general increase in anxiety and negative feelings, but a decrease in positive feelings after stress induction. This effect was observed after the start of the clips and persisted until the end of the clips.

### Cortisol

The cortisol analysis revealed significant main effects of emotional condition ([F (1, 69) = 10.25, ***p*** **=** **0.002**, η^2^_p_ = 0.129]) and time point ([F (2.85, 196.81) = 5.893, ***p*** **<** **0.001,** η^2^_p_ = 0.08]), while no significant emotional condition x time interaction was observed [F (2.73, 188.48) = 2.122, *p* = 0.105**,** η^2^_p_ = 0.03]. Post-hoc tests (shown in Fig. 2d) revealed an increase in Cortisol levels for both, control and stress conditions at time point 4 as compared to time point 1 (baseline). Post hoc analyses also revealed a significant increase of cortisol in the stress condition compared to the control condition at time points 3 and 4.

### Cytokines

We analyzed all targeted cytokines via ANOVAs, and for all cytokines, the respective ANOVAs revealed a significant main effect of time, but not of emotional condition, and the respective interactions (for the ANOVA results, refer to Table 1). The post hoc analysis conducted for the factor time revealed a common pattern of increased cytokine concentrations in both control and stress conditions during the time course of the experiments. Significant changes in cytokine concentrations relative to baseline in both, control and stress conditions are marked in Fig. 3.

**Table 1:** Main and interaction effects of the ANOVAs conducted for all targeted cytokines. Significant p-values are marked in bold.

	*Main effect of Emotional condition*				*Main effect of time*				*Interaction effect*			
	*df*	*F value*	*p value*	η^2^_p_	*df*	*F value*	*p value*	η^2^_p_	*df*	*F value*	*p value*	η^2^_p_
**IFN-γ**	1 (71)	.801	.374	.011	3.282 (233.03)	15.034	**<0.001**	.175	3.11 (220.93)	1.15	.329	.016
**IL-1β**	1 (71)	3.340	.072	.045	3.46 (245.74)	9.37	**<0.001**	.117	3.62 (257)	1.19	.316	.016
**IL-6**	1 (71)	2.030	.159	.028	3.36 (238.65)	10.66	**<0.001**	.131	3.37 (239.38)	1.23	.299	.017
**IL-4**	1 (71)	.365	.548	.005	3.06 (217.32)	21.09	**<0.001**	.229	3.18 (225.78)	1.43	.232	.020
**IL-8**	1 (71)	1.183	.280	.016	1.63 (115.48)	3.70	**<0.001**	.05	2.39 (169.98)	.414	.698	.006
**TNFα**	1 (71)	.559	.457	.008	3.23 (229.58)	13.73	**<0.001**	.162	3.32 (236.08)	.901	.45	.013

### HR

For HR analysis, the ANOVA revealed no significant main effect of emotional condition [F (1, 76) = 0.132, *p* = 0.718, η^2^_p_ = 0.002], but a significant main effect of time [F (8.44, 641.67) = 12.63, ***p*** **<** **0.001**, η^2^_p_ = 0.143], and a significant emotional condition*time interaction [F (7.6, 578.17) = 3.85, ***p*** **<** **0.001**, η^2^_p_ = 0.048]. Pair-wise post hoc comparisons (shown in Fig. 4a.) for time points in different emotional conditions revealed a significant increase of HR as compared to baseline (t1) in both conditions during the clips. Post-hoc comparisons between the control and stress conditions for each time point revealed an initial increase of heart rate in the stress condition at the start of clip 1 (t5), and then a deceleration leading to reversal of the difference towards the end of clip 1 (t7). HR differences then remained non-significant between control and stress conditions for the remaining time points.

### HRV

For HRV analysis, the ANOVA results show significant main effects of emotional condition [F (1, 76) = 5.412, ***p*** **=** **0.023**, η^2^_p_ = 0.066], and time [F (9.55, 725.94) = 11.55, ***p*** **<** **0.001**, η^2^_p_ = 0.132], and a trendwise interaction effect of emotional condition*time [F (9.03, 686.55) = 1.75, ***p*** **=** **0.074**, η^2^_p_ = 0.023]. The pair-wise post hoc comparisons (shown in Fig. 4b) conducted for each time point vs baseline for each intervention condition separately showed a significant decrease of HRV as compared to baseline (t1) in the stress condition (t2, t7–10, t12–17), while in the control condition a significant increase of HRV was observed for single time points (t11, t17, t19). Post-hoc comparisons between control and stress conditions for each time point showed a significantly decreased HRV for the stress condition during Clip 3 (t11-t13), the start of Clip 2 (t8), and Clip 4 (t14).

### Correlations between stress markers

To test if cytokine responses were related to other stress responses, we conducted Pearson’s bivariate correlations between cytokine area under the curve (AUCi) on the one hand, and cortisol AUCi as well as the AUCi values of HR and HRV on the other for both, control and stress conditions, as in a previous study (Larra et al., 2023). Negative correlations between IL-4 and cortisol (*r* = −0.233, *p* = 0.049) and IFN-γ and cortisol (*r*=−0.262, *p* = 0.026) emerged in the stress condition only.

### EEG power analysis

for EEG power calculation during clip presentation, we analyzed all 62 EEG electrodes at different frequency ranges (θ, α, β (low), β (high), γ (low), and γ (high)). We first analyzed each clip individually and later combined the EEG over all clips (clips 1–4). In each analysis we compared the stress clips with the respective control clips. We observed clusters in the Theta, Alpha, low Beta, low Gamma, and high Gamma range. Power was decreased in Theta, Alpha, and low Beta frequencies in each clip and also over all clips combined in the stress condition as compared to the control condition. Alpha power was lower in most of the electrodes, while for Theta the respective clusters of reduced power were situated over frontal, temporal, and occipital regions. For low Beta frequencies, specific clusters of stress-related reduced power were identified in frontal, central, and parietal areas. In the Gamma range, power was significantly larger in clips 2 and 4 for low Gamma, and only in clip 4 for high Gamma oscillations. For both, low and high Gamma, respective clusters of stress-related larger Gamma power were identified over occipital, temporal, and parietal regions. For the exact location and size of these clusters refer to Fig. 5. Further cluster details for each analysis can be found in the supplementary table 2.

For EEG power during the RS for control and stress conditions, we compared RS 1 (RS1) and 2 (RS2), before and after movie clip exposure for both, control and stress conditions in eyes open (EO) and eyes closed (EC) states for each frequency band. We plotted the topographic difference by comparing RS2 and RS1 (RS2-RS1) for control and stress conditions for both, EO and EC states for each frequency band. We did not identify any significant changes in the control condition for EO and EC states. In the stress condition, we found however significant changes in both, EO and EC states. In the EO state, a significant power increase in high frequency bands, including high Beta, with clusters in parietal and right fronto-temporal areas, low Gamma, and high Gamma, with clusters over almost the whole scalp, was revealed. In the EC state, a significant power increase was identified for all predefined frequency bands with clusters spanning the whole scalp. For the exact location and size of clusters refer to Fig. 7a, and supplementary table2.

To analyze group differences during RS, we compared RS activity between control and stress conditions during RS1 and RS2 for both, EO and EC states for each frequency band. We plotted the topographical difference by comparing stress and control conditions during RS (stress-control). We found no significant difference between control and stress conditions during RS1 for the EC state, while for EO state, lower power was revealed in the high Beta range in the stress condition, with a cluster over the right frontal, and left parietal regions. For RS2, in the EC state increased power was revealed in the stress condition for high Gamma oscillations. A respective cluster was identified over frontal, temporal and parietal regions. No significant difference between intervention conditions was seen in the EO state in RS2. For exact location and size of all clusters refer to supplementary figure S1 a.

### Connectivity

The connectivity analysis during movie clip presentation was performed for all electrode pairs via the coherence method. The resulting matrix of 62 × 62 connectivity pairs (excluding self- and mirrored connections) was contrasted between control and stress conditions for each clip, and the resulting significant connections are plotted in Fig. 6. The analysis was done for all targeted frequency ranges separately, as described for the power analysis. The largest connectivity differences were revealed for Theta, low Beta, high Beta, and low Gamma bands, while only minor differences were present in Alpha and high Gamma frequency bands. We observed the most prominent changes in Clip 4. Here connectivity decreased in the Theta band in the stress vs control condition with respect to fronto–posterior, and fronto-central to posterior connections. In the low Beta frequency band, a relatively decreased connectivity in the stress condition for left fronto-central and right posterior-occipital regions was revealed. In the high Beta and low Gamma frequency bands, similar changes were observed in the stress condition as compared to control, with decreased connections between fronto–posterior regions, but in contrast, increased connections between right temporal and parieto-occipital areas were revealed. Similar changes were revealed in the high Beta frequency band for Clip 2 as well. Please refer to Fig. 6 for a detailed presentation of all significant connectivity changes (for a list of all connections refer to the supplementary excel file, for an electrode to electrode statistical matrix for each analysis please refer to the supplementary material, Clip Connectivity matrices.

We furthermore performed a connectivity analysis for the RS as described above for the clip connectivity analysis. We found connectivity alterations in RS2 compared to RS1 (Fig. 7). We compared RS1 and RS2 for both, control and stress conditions in EO and EC states, as in the respective power analysis above. We did not identify any significant connectivity differences between RS1 and RS2 in the control condition for EO and EC states. For the stress condition, in the EO state we identified significant changes in the Theta, low Gamma, and high Gamma frequency bands between RS1 and RS2. Specifically, an increased connectivity between left frontal (F6) and parieto-occipital regions was observed. In the EC state of the stress condition, significant connectivity increases in the Theta, Alpha, and low Beta frequency ranges emerged, however only few changes were observed in high Beta, low Gamma and high Gamma. Specifically, in the Theta frequency range, a connectivity increase was observed for within-frontal connections and left fronto - occipital connections. In the Alpha range, a connectivity increase was significant for within-frontal and posterior-central and left-parieto-occipital connections. In the low Beta frequency range, a connectivity increase was observed only for centro-posterior and left-parieto-occipital connections. In the Gamma frequency range, few increased connections within frontal and right parietal regions were observed. Comparing RS connectivity for control and stress conditions within RS1 and RS2, we found no significant connectivity differences in both EO and EC states. Please refer to Fig. 7 and S1b (for a list of all connections refer to the supplementary excel file; for an electrode to electrode statistical matrix for each analysis please refer to the supplementary material – RS connectivity matrices.

## Discussion

The goal of the present study was to investigate the efficacy of stress induction in healthy human adults through aversive video clips and to explore the dynamics of stress effects on emotion, brain, and body physiology by recording data from multiple modalities. We aimed to replicate previous findings from studies that employed similar stress induction paradigms and to explore previously uncharted territories within the context of this stress induction paradigm (Noack et al., 2019).

In this study, we employed a predetermined sequence of clips for both control and stress conditions for several reasons. First, many previous studies that applied the AVP to induce stress have employed a fixed sequence of clips. Since our objective was to evaluate the effectiveness of the AVP in inducing acute stress as a paradigm, we aimed to adopt a methodology that closely resembles prior research (Gärtner et al., 2014; Qin et al., 2009). Second, we maintained the order of the clips to preserve the integrity of the storyline. Given that the film’s narrative is innately distressing, the initial context given enhanced the participants’ immersion and emotional engagement. This is supported by the narrative transportation theory, which suggests that providing a coherent context and storyline prior to viewing enhances affective responses to the content of a movie (Green & Appel, 2024). Finally, in contrast to earlier studies that utilized predetermined sequences and inserted tasks between clips, our research concentrated on exploring the combined, uninterrupted impact of viewing distressing scenes within the narrative context. This approach yielded a more synergistic, context-driven emotional response to the stressful clips.

### Subjective and cardiac results

Our main results indicate a significant increase in negative affect scores and a reduction in positive affect scores following exposure to aversive video clips, along with increased anxiety scores as measured by STAI. Physiological measurements during the stress condition revealed a decrease in HRV during the movie clips as compared to the control condition and an initial increase in HR, which later decelerated again relative to this increase and became similar to HR in the control condition. Uncertainty of events can lead to increased HR (Corcoran et al., 2021)**,** in accordance with the initial HR increase during the stress condition, as participants were aware about the start of the clips. Previous studies using movie clips as affective stimuli have also shown that movies depicting unpleasant or pleasant stimuli lead to HR deceleration with higher deceleration for aversive clips (Codispoti et al., 2008; Hiser et al., 2021), which is also in general accordance with the results of the present study, where an initial increase of HR was followed by a relative decrease, indicative of enhanced sensory processing and attention during the clips (Corcoran et al., 2021). The decrease of HRV during clip presentation in the stress condition is likely caused by reduced parasympathetic activation due to clip presentation and thus a reduced vagal impact on the cardiac rhythm (Laborde et al., 2017). Thus, in the present study, the clips intended to induce stress led to increased anxiety and negative emotions, and reduced HRV, and thus psychological and physiological alterations indicative of stress (Hogenelst et al., 2024)**.**

### Hormonal (cortisol and cytokines) results

We also observed elevated salivary cortisol levels, suggesting activation of the HPA axis, in accordance with previous studies using aversive video clips (Noack et al., 2019; Qin et al., 2009; Takai et al., 2004). Furthermore, the study explored the dynamics of cytokine-mediated immune responses to acute stress using the AVP. While psychological stress, in general, is known to influence cytokine activity (Russell & Lightman, 2019), limited research is available on the immediate cytokine dynamics following acute stress induction. Specifically, no respective study using the AVP is available. We observed a significant increase in the levels of all explored cytokines during the experiment in both control and stress conditions, hinting at more general experimental or contextual rather than AVP-specific effects. This result is similar to a previous study, which also reported similar cytokine enhancements in both stress and control conditions during the Cold Pressor Test (CPT) (Larra et al., 2023). Although the AVP shows SNS activation, it does not involve substantial physical activity, and its control session is highly similar in structure and engagement level. Hence, the lack of physical or task-related differences between the emotional conditions could explain the similar cytokine dynamics observed in both conditions. Therefore, our current cytokine findings do not support a stress-specific immune response elicited by the AVP.

However, a few points should be taken into account when considering these findings. First, cytokine analyses using saliva have several relevant limitations, including the impact of oral health, transport of cytokines to saliva, and the low correlation between immune levels in saliva and blood (Slavish et al., 2015). Second, even though in one respective review paper it was shown that acute stress led to an increase of inflammatory cytokines, this increase was mainly prominent in studies comparing an active stress session (mainly including a physical stressor) with a rest condition (without any physical activity) which suggests a strong physical activity-dependency of cytokine levels. The latter aspect is lacking in the AVP paradigm (Slavish et al., 2015).

With respect to the association of cytokines and cortisol, we identified similar to Larra et al. a significant negative correlation between cytokine and cortisol levels only in the stress condition, and the cytokines for which we found these correlations were IFN-γ and IL-4, hinting at a possible stressor-specific cortisol-cytokine interaction (Larra et al., 2023).

### EEG power changes

Concerning the effects of the stress induction protocol on oscillatory brain activity, we analyzed absolute power differences between stress and control conditions in multiple frequency bands during clip presentation, and in RS before and after clip presentation. Since stress exerts its effects on a broad range of EEG frequencies, analyzing multiple frequency bands is required to receive a complete picture (Katmah et al., 2021).

In the power analysis conducted for the EEG during clip exposure, we consistently observed a power decrease within the low-frequency range (4–15 Hz) across all stress-inducing clips, but a power increase within the high-frequency range (32–80 Hz) for specific clips. In the Theta range, this reduction was mainly seen in frontal and temporal areas. Alpha reduction, another common EEG alteration during stress, was visible for all electrodes in all clips. In the Beta range, only low Beta (12–15 Hz) showed a decrease over central, frontal, and parietal regions in all clips, while high Beta remained unchanged. In the Gamma and high Gamma frequency ranges, we observed a power increase during presentation of clips 2 and 4, most prominently over occipital, temporal, frontal, and parietal regions.

Comparable alterations of low-frequency oscillations during stress exposure have also been reported in previous studies, where stress led to a reduction in frontal Theta (Gärtner et al., 2014), Alpha (Katmah et al., 2021; Vanhollebeke et al., 2022), and low Beta (Marzbani et al., 2016; Thompson & Thompson, 2007) frequency bands. Low-frequency oscillations are known to be involved in the top-down processing of information (Keil & Senkowski, 2018; Xiong et al., 2023). Previous research has furthermore highlighted a role of Gamma activity in negative emotions, such as processing negative faces (Luo et al., 2007) and in worry and generalized anxiety (Oathes et al., 2008). Increased high-frequency power in similar areas as in the present study was also reported in a previous study with aversive affective pictures (Müller et al., 1999). Previous research has moreover suggested an involvement of Gamma-range activity in bottom-up processing (Riddle et al., 2019).

In the analysis of RS power before and after stress induction, a notable difference emerged solely for the stress intervention condition. For the EO state, an enhancement of high-frequency band power was evident after presentation of the stress clips, particularly in high Beta, low Gamma, and high Gamma bands. In the EC state, a significant power increase spanned all frequency bands of interest. Comparing RS EEG power for control and stress conditions after presentation of the movie clips unveiled selectively enhanced High Gamma activity in the stress group. Increased power in all frequency bands during the RS has been reported during enhanced uncertainty and anxiety (Knyazev et al., 2005) and a rise in high-frequency power (Beta, Gamma) during the RS has also been associated with increased worry and arousal (Oathes et al., 2008; Ribas et al., 2018).

### EEG connectivity changes

In the connectivity analysis conducted for the EEG during clip presentation, notable connectivity differences emerged in Theta, low Beta, high Beta, low Gamma, and high Gamma ranges. Connectivity between fronto-central and parietal regions was reduced for all these frequency bands during stress induction compared to the control condition, particularly during Clip 4, indicating a potential influence of clip content. Additionally, during the Clip 4 presentation and when all clips were combined, we observed an increase in connectivity between the right temporal and posterior areas for high Beta, low Gamma, and high Gamma frequency ranges. In Clip 2, we found such connectivity differences only for the high Beta range.

Fronto-posterior connections have been implicated in regulatory control processes and are thought to underlie top-down modulation of perceptual areas by prefrontal regions (Buschman & Miller, 2007). Previous EEG-based emotion classification studies have demonstrated that connectivity patterns in the Beta and Gamma frequency ranges can reliably distinguish stress or aversive emotional states (Mishra et al., 2022; Yang et al., 2020). Supporting this, studies have reported reduced Beta-band coherence between frontal and posterior regions during negative emotional processing (Hao et al., 2019; Reiser et al., 2012), as well as in patients with generalized anxiety disorder (GAD) (Chu et al., 2025; Wang et al., 2025). These results are interpreted as reflecting reduced regulatory engagement or altered integration across distributed networks, resulting in a loss of top-down control over perceptual areas. The right temporal lobe has been implicated in the processing of aversive and emotional stimuli (Phan et al., 2004; Xie et al., 2025), and the parietal cortex has also been associated with emotional salience and sensory integration (Gray et al., 2002; He et al., 2021).

In the present study, we observed decreased frontal–posterior connectivity alongside increased connectivity between right temporal and posterior regions during the AVP. While our sensor-level analysis cannot directly infer the directionality or source of these effects, this pattern may reflect altered large-scale network coordination under stress, a phenomenon similar to that observed in previous studies.

The observed reduction in low-frequency power and diminished fronto-posterior connectivity is consistent with theories of stress-induced disruption of regulatory control (De Kloet et al., 2019; Joyce et al., 2025; McRae et al., 2012), and also seen in anxiety disorders (Sylvester et al., 2012). In parallel, the increased high-frequency activity and connectivity between right temporal and posterior regions could reflect heightened sensitivity to bottom-up sensory-affective inputs. However, given the spatial and directional limitations of sensor-level EEG, these interpretations should be considered cautiously and warrant confirmation through future source-level or functional magnetic resonance imaging (fMRI) analyses.

No connectivity differences were observed between stress and control clips in both RS. However, after presentation of the stress-inducing clips in the EC state, increased Theta connectivity in the right frontal area and between left fronto-central and occipital regions was observed as compared to pre-intervention RS (baseline). Moreover, in the Alpha frequency range, connectivity rose within the frontal area and between parietal and left temporal regions as compared to baseline.

Moreover, enhanced low frequency (Theta and Alpha) connectivity in RS has been reported in dysphoria patients associated with enhanced rumination and self-focus (Dell’Acqua et al., 2021) as well as in generalized social anxiety disorder (gSAD) patients (Xing et al., 2017). Thus, the results of the present study are in accordance with a state of increased ruminative thinking and arousal after aversive video clip presentation, which was not observed after the neutral clips.

### Comparison with psychosocial stressors

In essence, we found that the AVP as a stress induction paradigm effectively elicits activation of the HPA axis and the SNS system, making it a reliable method for inducing stress. In comparison to other established psychosocial stress induction paradigms, such as the TSST or MIST, the AVP has several practical and conceptual advantages. It requires minimal staff, setup, and training, facilitating easy application in various imaging and lab settings, which makes it a cost-effective option for stress induction, especially for resource-limited studies. Unlike the TSST and MIST, which focus on psychosocial stress through social evaluations and performance pressures, the AVP generates psychological stress by placing participants in emotionally charged, real-world-like aversive situations, enhancing its ecological validity. Additionally, the AVP allows researchers greater experimental flexibility, enabling the incorporation of pre- or post-stress cognitive or physiological assessments within a single session. Importantly, while repeated use of the TSST often results in quick habituation (Frisch et al., 2015), the AVP allows repeated measures designs by varying the aversive clips, thus preserving novelty and reducing desensitization. These factors make the AVP a scalable and adaptable tool for stress research, particularly when combining multimodal imaging or behavioral assessments.

## Limitations and future directions

While this study identified clear psychological and physiological effects of the AVP protocol indicative of its suitability as a stress-inducing tool, some limitations should be taken into account. The stress induction procedure used in this study includes factors beyond pure stress, such as disgust, potentially influencing the observed responses. The consistent clip sequence, while maintaining paradigm continuity, introduces potential confounding effects from specific clip content interactions with neural dynamics. Potential future studies should aim to counterbalance and randomize the clip sequences, to disentangle specific or unspecific clip content effects and better isolate stress responses. Additionally, the inclusion of only males limits the generalizability of findings across sexes. We recruited only males since previous studies have shown that the effects of acute stress can be influenced by gender, menstrual cycle phases, and use of contraceptives in females (Goldfarb et al., 2019; Jentsch et al., 2022; Kajantie & Phillips, 2006). Additionally, the movie “Irreversible”, which was used for the stress clips, always has a male as the perpetrator of violence (clips show only male-to-female and male-to-male violence), which might bias stress effects based on gender of the actor who commits the violence, and participant gender. To address these limitations, future studies should diversify participant populations, including both sexes and add clips from diverse movies with more balanced perpetrators based on gender. The conduction of a working memory task after the clips also raises the possibility of interferences between task-induced anxiety and clip effects for the data obtained after clip presentation, warranting further investigation in future research. AVP as a stress paradigm can also be easily integrated into exploring stress-reactivity in clinical populations, especially in people suffering from anxiety disorders or PTSD. Exposure to such stressful scenes (especially those with personal relevance to the patients) will help to understand the ongoing neural dynamics in a clinical population. Comparing stress responses to the AVP between healthy controls and clinical populations will also help to identify potential stress markers, which may serve as targets for neuromodulatory interventions such as NIBS. Accordingly, we employed these clips to investigate the effects of AVP-induced stress on working memory performance, where stimulation of the ventromedial prefrontal cortex (vmPFC) was found to mitigate working memory deficits and reduce stress reactivity (Roy et al., 2025).

## Conclusions

This study aimed to assess the stress-inducing efficacy of the AVP in healthy humans. We found that exposure to aversive clips led to increased negative affect, reduced positive affect, and elevated anxiety scores. Physiologically, salivary cortisol levels increased, and we observed altered HR and HRV dynamics, all of which indicated successful stress induction based on HPA and SNS markers. The cytokine analysis showed elevated levels during both stress and control conditions, suggesting a nonspecific immune response to this paradigm. The EEG during the clips revealed reductions in low-frequency power, increased high-frequency power, and altered connectivity patterns during stress induction, indicating potential disruptions in top-down control and activation of bottom-up processing. RS EEG showed increased power across all frequency bands after stress induction, suggesting sustained effects of stress on neural activity even after presentation of the stressor. Our results provide a multimodal understanding of stress responses elicited by the AVP, which may be useful for future research aimed at eliciting a more psychological stress response.

## Methods

### Participants

Seventy-eight healthy, right-handed male non-smokers aged between 18 and 40 years participated in the study (*M* = 25, SD = ±4.16). A medical check before the experiment made sure that the participants did not suffer from any chronic or acute disease, did not have any psychiatric or neurological disorder, did not suffer from any inflammation of the mouth, were not taking any central nervous system (CNS) acting medication, did not consume >0.2 L wine or 0.5 L beer every day, did not work at night or changing shifts during the timeframe of the experiment, nor had any implants or devices in their body. Participants with a history of epilepsy or traumatic brain injury were also excluded. In a pre-experimental phone interview, it was ensured that the participants did not have any major physical or emotional trauma and that they had no habit of watching extremely violent movies. Before the experimental session, participants were instructed to refrain from consuming any coffee or energy drinks for 2 h prior to the experiment. Additionally, participants were asked if they had seen any particularly violent French movies to rule out the possibility of prior familiarity with the clips. We recruited subjects by putting up flyers around the nearby university campuses. Additionally, we utilized university mailing lists and social media postings to recruit participants for the study. Participants were appropriately financially compensated in accordance with local hourly rates.

The study was approved by the Ethics Committee of the Leibniz Research Centre for Working Environment and Human Factors at TU Dortmund (IfADo) [Ethics approval number – 197; File number – 2021/197/2021–03–16] and aligns with the Declaration of Helsinki. Participants were informed about their right to withdraw from the experiment at any time, and written informed consent was obtained from all participants before the start of the experiments.

### Experimental procedure

Participants underwent a three-session experimental protocol, starting with a practice session aimed at reducing unspecific experiment- and context-related stress, conducting a medical check, and training of the participants in a working memory task, which will be reported in detail in another publication. The medical check was a face-to-face evaluation performed by a medical doctor at the research institute (IfADo). During the practice session, we also showed a still image from the stress movie and asked if they knew the scenes from before to rule out the possibility of prior exposure to the movie clips. Subsequently, two experimental sessions—control and stress conditions were administered, with randomized and counterbalanced session order based on a random pre-generated order (https://damienmasson.com/tools/latin_square/). All experiments were conducted between 12 pm. and 6 pm. to control for endogenous cortisol activity. One experimental session was done within a week after the first (practice) session, with at least an interval of a day. Between the two experimental sessions (control and stress), there was a minimum one-week washout period. All sessions were done on different days. The total duration of the experimental sessions was around 3 h from the arrival of the participants until they left, and 1.5 h for the practice session.

**Fig. 1:**
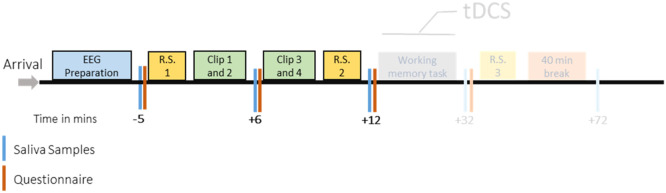
Experimental procedures. After arrival of the participants, the EEG cap was prepared. Then RS EEG was conducted (R.S.), with 2 min Eyes Open (EO) and 2 min Eyes Closed (EC) EEG. Clips 1–2, and Clips 3–4 denote the movie clips shown (aversive or neutral, according to the control or stress session). Blue bars show the time points of saliva (cortisol, cytokines) sampling and red bars show time points when STAI and PANAS were administered. The time points for saliva sampling and questionnaire conduction in relation to the start of the intervention (clip presentation) are shown below the bar in mins. HR (using bipolar electrodes positioned on chest) and EEG were recorded during the whole experiment starting from R.S.1 to R.S.3. The procedure was identical for control and stress sessions except for the kind of movie clips shown. The latter part of the experiment, including tDCS and working memory task performance, is shown in opaque color and will be reported in detail elsewhere as it is out of scope of the topic of this article.

For the experimental sessions, participants arrived one hour prior to EEG scanning for electrode placement. The experiment began with baseline cortisol and questionnaire assessments, followed by an EEG RS recording (2 min with EO and 2 min with EC). Subsequently, the movie clips were presented on a monitor, with 30 cm eye to screen distance and audio output provided via standard in-ear earphones. Saliva samples were obtained before starting the experiment as baseline and then after the first two movie clips, and then the remaining two clips were shown. Afterwards, another set of saliva samples and questionnaires were collected, followed by the final RS EEG recording, as described above. The complete experiment included two parts: the first part is described above, and a second part, in which working memory performance was assessed via an n-back task combined with non-invasive brain stimulation, will be described elsewhere. For an overview, please refer to Fig. 1.

**Fig. 2:**
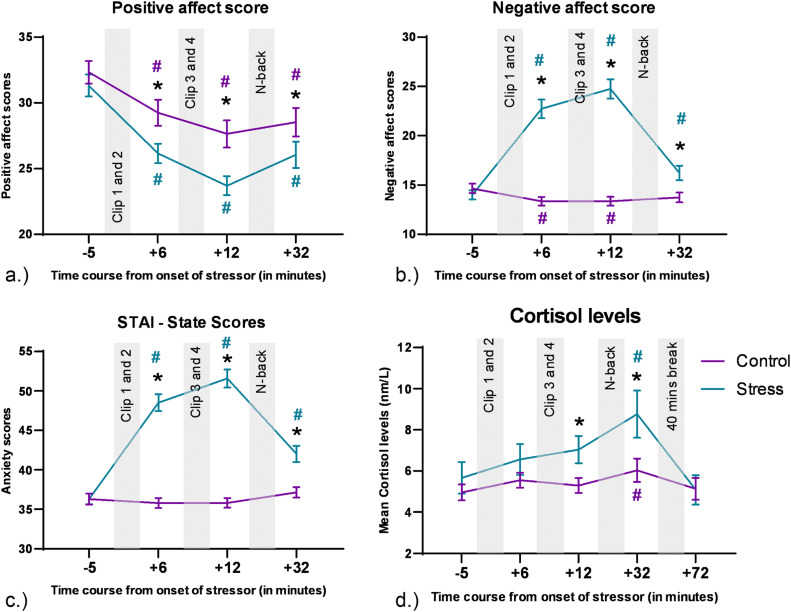
Behavioural and physiological data during stress exposure. a.) Positive affect scores during the experiment. b.) Negative affect scores during the experiment. c.) STAI-S scores during the experiment. For these subjective scores, the y axis denotes the scores in the questionnaire and the x axis shows the time course relative to the start of the movie clips. The last time point of subjective data refers to the time point after conduction of the N-back task, which is out of scope of this paper, and these changes might be also influenced by the task. d.) For cortisol levels, the y axis displays mean cortisol levels (nm/l), and the x axis shows the time course relative to the start of the movie clips. Error bars denote ± SEM. Asterisks (*) denote significant differences (critical p-value ≤0.05) between intervention conditions for each time point. The hash symbol (#) denotes significant differences (critical p-value ≤0.05) between time points during intervention and baseline (time point 1) in each condition. The respective color of (#) indicates the respective intervention condition. The last two time points of the cortisol measures were conducted after N-back task performance, which is out of scope of this paper.

### Stress induction procedure

Stress was induced using aversive video clips from the movie "Irreversible" by Gaspar Noe, while control condition clips were extracted from the movie "Comment j'ai tué mon père" by A. Fontaine. Each clip had a duration of 3 min, with four clips presented in a predetermined sequence. Control and stress condition clips were carefully matched for duration, luminance, and presence of humans, differing only in violence levels. The first two stress clips depicted violence against a female, while the last two depicted violence against a male. The first two control clips involved a party scene, and the last two clips involved long shots of people walking. Before the start of each condition, we provided the following context to the participants. For the stress condition: “The scenes are meant to be quite stressful, dizzying, and uncontrollable. The man in the scene who gets beaten up is the lover of the girl who also gets beaten up. The guy who does the crime remains free”. For the control condition: “The scenes are meant to be slow, relaxing, and neutral. The old man is the father of the young man. They both have complicated relationships. The girl in the clips is the wife of the young man. For exact details regarding each clip’s content and timestamp from the original movie please refer to the supplementary sheet, Sections 1 and 2. Both movies were in French language without subtitles. Only two of the included participants had French language proficiency. Before viewing, participants received a brief introductory text instructing them to watch the movie clips attentively from an eye-witness perspective.

### Subjective data

Two questionnaires were administered at various time points during the experiment: the PANAS (Krohne et al., 1996; Watson et al., 1988) and the STAI-S (Laux et al., 1981; Spielberger et al., 1983). The PANAS assessed positive and negative feelings and included 20 questions, which had to be rated on a 5-point scale ranging from 1 (not at all) to 5 (very much), while the STAI-S evaluated state anxiety levels, also included 20 questions which had to be rated on a scale ranging from 1 (not at all) to 4 (very much). Participants were given the choice to fill in the questionnaires in either German or English language based on their personal preference. Data were collected using the online Sosci survey platform (Leiner, 2022) and subsequently analyzed using custom MATLAB scripts.

### HR and HRV data

HR was continuously monitored during the experiment using two bipolar electrodes connected to Bipolar inputs of a NeurOne JackBox (Neurone, Finland)— one electrode was attached below the right clavicle and the other electrode was attached above and left to the umbilicus. HR data were sampled at 2000 Hz and analyzed using HEPLAB (Perakakis, 2019), an EEGLAB plugin Delorme and Makeig (2004), along with custom-made MATLAB scripts. Inter-beat intervals (IBIs) were calculated, and HR was computed for each 1-minute interval during the experiment during RS and presentation of the movie clips (for details, refer to Fig. 1). HRV was determined using the Root Mean Square of Successive Differences (RMSSD) method (Shaffer & Ginsberg, 2017) via a custom-made MATLAB script for identical 1-minute bins as for HR.

### Cortisol

Saliva samples were collected at various time points (for exact time points and intervals refer to Fig. 1) during the experiment using Sarstedt Salivettes. Cortisol levels were analyzed using the Cortisol Saliva ELISA (TECAN/IBL International) with following the manufacturer's instructions. Absorbance at 450 nm was measured using a GloMax® Multimode Microplate Reader System (Promega). Cortisol data from only 70 participants were analyzed due to the limited availability of kits and insufficient saliva samples from some participants.

### Cytokines

Cytokine levels in saliva were measured twice per sample using the LEGENDplex Human Essential Immune Response panel (BioLegend), as described in previous studies (Larra et al., 2023; Maydych et al., 2018). Briefly, 10 μl saliva or standard control solution was added to a V-bottom 96 well plate mixed with 30 μl assay buffer and 10 μl beads and incubated for 2 h at room temperature (RT) on a shaker at 800 rpm. After washing with 200 µl wash buffer (provided in the kit), a 10 μl biotinylated detection antibody mix was added to the beads and incubated for 1 h at room temperature on a shaker at 800 rpm. Then, 10 μl PE-conjugated streptavidin was added, followed by an additional incubation for 30 min at RT on the shaker at 800 rpm. After two washes with wash buffer, the PE fluorescence intensity of the beads was measured on an LSRFortessa flow cytometer (BD Biosciences). Bead populations were identified by FSc/SSc features and fluorescence intensity in the APC channel. Approx. 200 beads per analyte were acquired. Data were analyzed using the LEGENDplexTM Data Analysis Software (VigeneTech). All samples from a participant were analyzed on the same day to avoid inter-assay variation. Cytokines targeted for analysis were Interferon-gamma (IFN-γ), Interleukin – 1Beta (IL-1β), Interleukin – 6 (IL-6), Interleukin – 4 (IL-4), Interleukin – 8 (IL-8), and Tumor necrosis factor - alpha (TNFα). We selected this profile of six essential cytokines to obtain a broad overview of stress effects on cytokines by targeting a mix of pro-inflammatory (IFN-γ, IL8, IL-1β, TNFα, and IL-6) and anti-inflammatory (IL-4) cytokines. These cytokines have been obtained in previous studies exploring the effects of stress on cytokines (Larra et al., 2023; Maydych et al., 2018; Slavish et al., 2015) . Cytokine data from 70 participants were analyzed due to the reasons given above.

### EEG data acquisition and processing

EEG was recorded during RS and during movie clip presentation against a reference electrode placed over the left mastoid, using sintered Ag-AgCl electrodes at 62 positions in accordance with the international 10–20 EEG System (NeurOne, Finland). Electrode impedance was monitored and kept below 10 kOhm throughout the experiment. The sampling rate was 2000 Hz at an analog-to-digital precision of 24 bits. Offline EEG analysis was done using EEGLAB (v2022.1) (Delorme & Makeig, 2004), Brain Vision Analyzer (Version 2.2.0, Brain Products GmbH, Gilching, Germany), Fieldtrip (Oostenveld et al., 2011), and custom MATLAB commands.

There were four movie clips, each lasting for 3 min. RS was recorded for 2 min for EO followed by 2 min for EC, keeping the order the same for all participants. Prior research has demonstrated that a 2-minute RS EEG recording can yield reliable results when the recording protocol and order are consistent across participants (Barry et al., 2007; van Diessen et al., 2015). Moreover, a similar RS duration in a previous study from our group also produced reliable findings (Salehinejad et al., 2022).

Data preprocessing was identical for all datasets and is described in detail inFig. 8. We used the Cleanline plugin to remove line noise (Mullen, 2012). We also used the automatic artifact removal (AAR) plugin of EEGLAB to remove eye and muscle activity (De Clercq et al., 2006; Gómez-Herrero et al., 2006). Additionally, we used the IClabel plugin to automatically label IC decompositions, which are suspected to be noise (Pion-Tonachini et al., 2019).

**Fig. 3:**
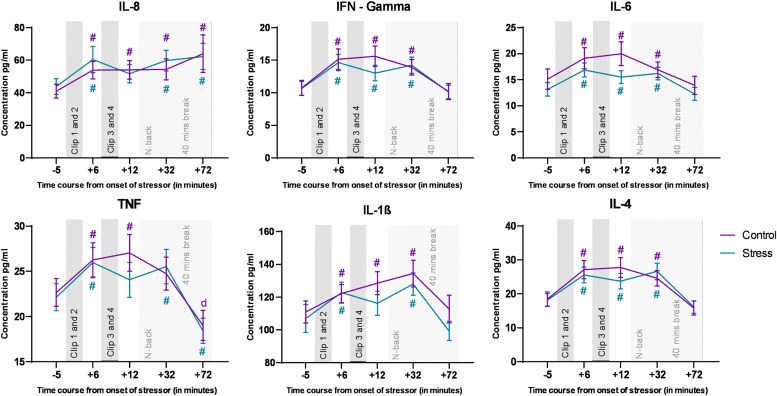
Cytokine levels during the experiment. The Y axis denotes cytokine concentration levels (pg/ml) and the x axis denotes time relative to onset of the movie clips. Hash symbols (#) denote significant differences (critical p-value ≤0.05) between baseline (time point 1) and the other time points within each condition. The respective color of (#) denotes the respective condition r. Error bars show ± SEM. The last two time points were obtained are after N-back task performance, and the dynamics of these time points might be influenced also by the task.

**Fig. 4:**
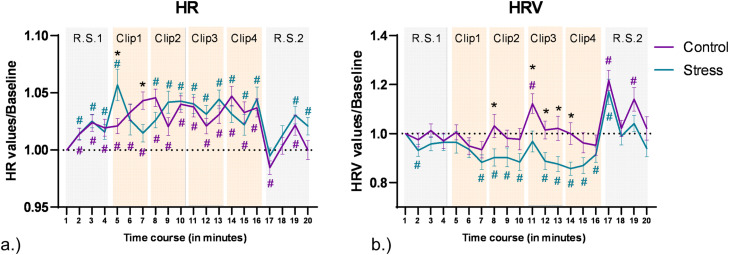
Cardiac data obtained during the experiment. a.) Heart rate during the experiment. The y axis denotes changes of heart rate from baseline, and the x axis denotes each minute of the experiment. Error bars denote ± SEM. Asterisks (*) denote significant differences (critical p-value ≤0.05) between conditions for each time point. The hash symbol (#) denotes significant differences (critical p-value ≤0.05) between time points and baseline (time point 1) in each condition. The respective color of (#) denotes the intervention condition. RS and clip time points are highlighted in the background with grey and orange colored watermarks (grey represents RS and orange clips clip presentation). b.) Heart rate variablility during the experiment. HRV was calculated based on RMSSD measures and plotted the same way as heart rate data.

**Fig. 5:**
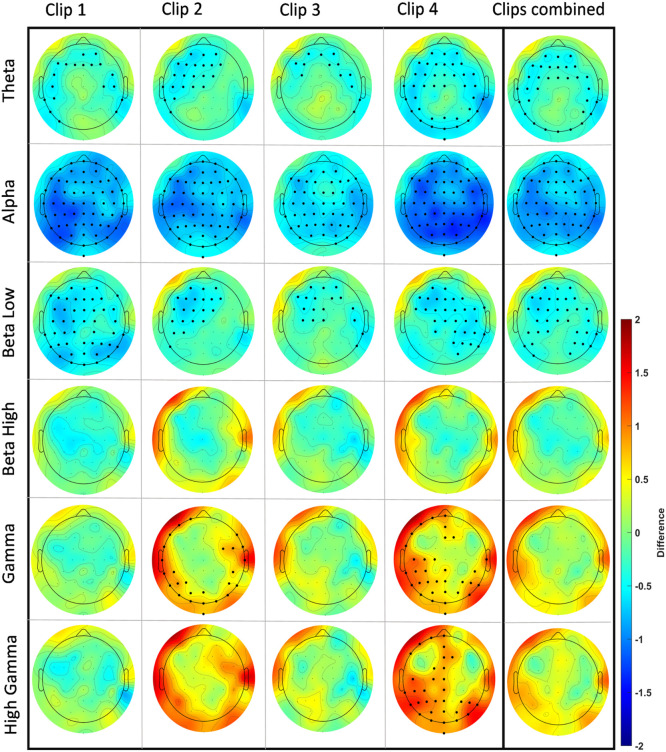
EEG power changes for each clip and all clips combined across the frequency bands of interest: Theta (4–8 Hz), Alpha (8–13 Hz), low Beta (13–15 Hz), high Beta (23–32 Hz), low Gamma (32–50 Hz), and high Gamma (50–80 Hz). Each topographical plot illustrates the mean difference (stress-control) between conditions. Red indicates a higher value, while blue indicates a lower value in the stress condition compared to the control intervention. To identify significant electrodes in each analysis, we employed Monte Carlo-based permutation statistics with cluster correction (see data analysis section). This permutation strategy involves permuting data to establish a data-driven null distribution. To address the issue of multiple comparisons, clusters (based on adjacency) were identified and compared with the null distribution using a critical p-value of < 0.01. Each EEG topographical plot features a cartoon head viewed from above, with the nasion at the top and the inion at the bottom. The color bar represents the range of difference values from 2 to −2. For a reference to a zoomed-in cartoon head with highlighted electrode locations and labels, please refer to supplementary figure 2.

**Fig. 6:**
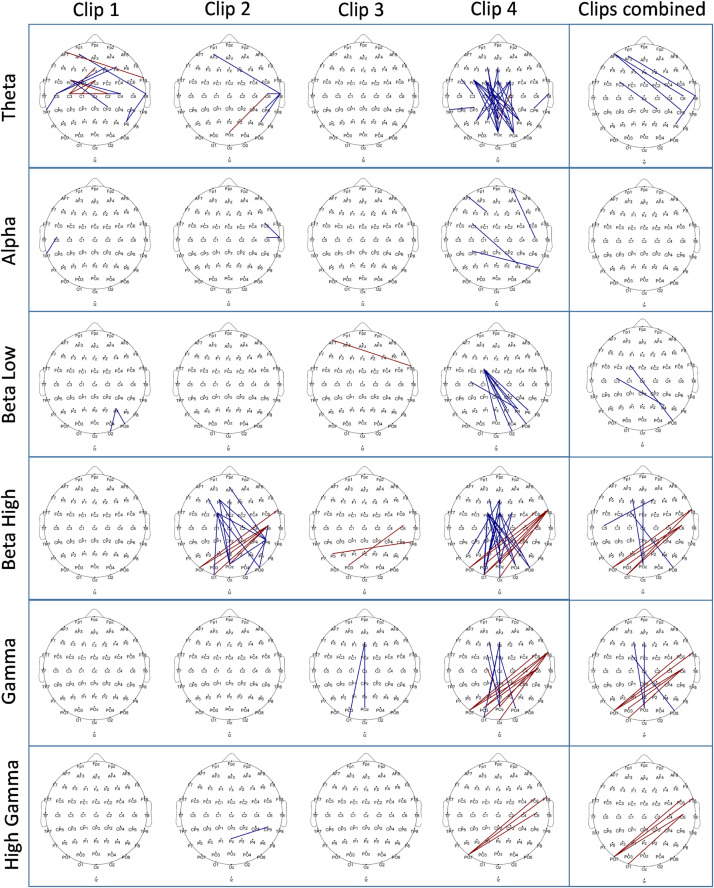
EEG connectivity differences between stress and control conditions are shown for each clip separately, as well as for all clips combined for each frequency band of interest (as in Fig. 5). Each topographical plot displays the significant connections (which survived after statistical thresholding) marked in two colors. Blue indicates a decrease in connectivity strength, while red indicates an increase in connectivity strength in the stress condition compared to the control condition. To identify the significant connections between the two conditions, the entire 62 × 62 connectivity matrix was analyzed using Monte Carlo-based permutation statistics with maximum statistics correction (see data analysis section), which identifies significant connections based on a distribution created by permuted data. The differences were marked as significant for a critical p-value of ≤0.05. The topographical plot illustrates significant connections using red or blue lines between electrode pairs. The cartoon head is depicted from above, with the nasion positioned at the top and the inion at the bottom. For a reference to a zoomed-in cartoon head with highlighted electrode locations and labels, please refer to supplementary figure 2.

**Fig. 7:**
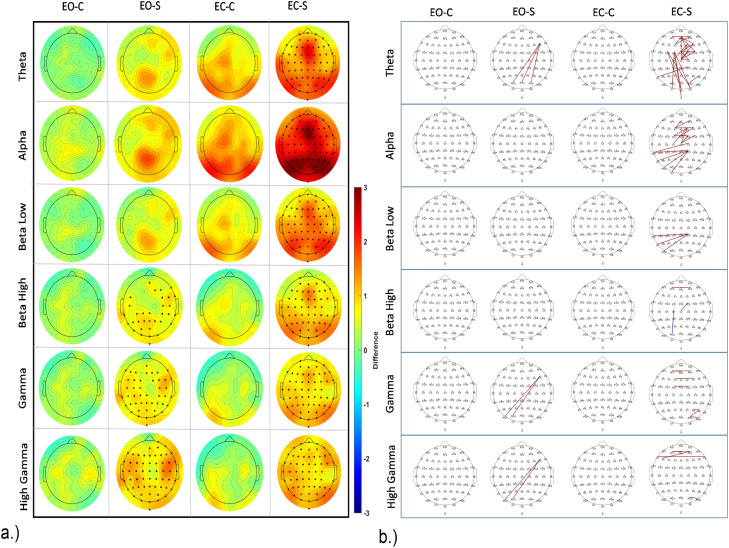
a.) EEG power changes during the RS for both EO and EC states. The topographical plot over the cartoon head (plotted similar to Fig. 5) displays mean power differences before and after intervention for EO and EC states, for stress and control conditions. Significant electrodes after corrections for multiple comparisons are marked in bold (as in Fig. 5). The color bar denotes the difference value range from 3 to −3. Red indicates higher power values in the post-intervention RS compared with pre-intervention, and blue indicates reduced power. b.) EEG connectivity differences during RS before and after intervention for both EO and EC states for control and stress conditions. Significant connections surviving multiple comparisons (with statistics as shown in Fig. 6) are marked in blue and red. Blue indicates a decrease of connectivity strength in the post-intervention RS (after clip presentation), and red denotes an increase of connectivity strength in the post-, as compared to the pre-intervention RS. The topographical plot shows significant connections on the cartoon head similar to Fig. 6. For a reference to a zoomed-in cartoon head with highlighted electrode locations and labels, please refer to the supplementary figure 2. EO—C denotes Eyes Open – Control, and EO-S denotes Eyes Open – Stress. EC—C denotes Eyes Closed – Control, and EC-S denotes Eyes Closed – Stress.

**Fig. 8:**
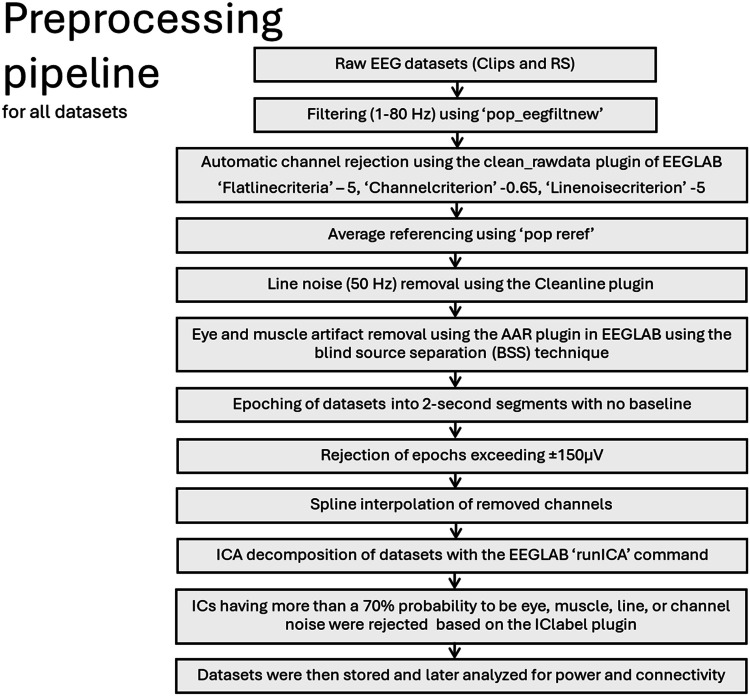
EEG preprocessing pipeline used for all datasets (movie clips and RS). We used EEGLAB (Delorme & Makeig, 2004) and custom MATLAB scripts for the analysis. Please refer to supplementary Table 3 for the average number of epochs and channels after rejection for each clip and RS (EO and EC) recordings.

### Power analysis

The power analysis employed the EEGLAB ‘STUDY’ function (Delorme & Makeig, 2004) and custom MATLAB scripts. The *spectopo* function was used for power estimation with a window size of 998 ms and overlap of 495 ms, and spectra were generated for all 62 channels and averaged within six frequency bands (θ – 4 to 8 Hz, α – 8 to 13 Hz, β (low) – 13 to 15 Hz, β (high) – 23 −32 Hz, γ (low) – 32 to 50 Hz, and γ (high) – 50–80 Hz). *Spectopo* uses the Pwelch method of the MATLAB signal processing toolbox for power estimation with overlapping hamming windows. The analysis was performed for all 62 electrodes and significant electrodes were identified for each analysis using Montecarlo-based permutation statistics with cluster correction (see data analysis). Topographical plots were generated using EEGLAB's *topoplot* function.

### Connectivity

Connectivity analysis was conducted using Fieldtrip (Oostenveld et al., 2011). Preprocessed data from EEGLAB were imported using the *‘ft_preprocessing’* command, frequency decomposition using the *’ft_freqanalysis’* command with the following input parameters [method = mtmfft, taper = dpss, output = fourier, foilim = [2 80], tapsmofrq = 2], and analyzed for connectivity by the *‘ft_connectivityanalysis’* using the coherence (coh) method.

Coherence as a measure for assessing functional connectivity between EEG signals is the most commonly used method to calculate linear dependencies between two channels in the frequency domain. This method was employed as it does not need any a priori hypothesis and previous research has shown that coherence as a measure can reliably differentiate stress indices as compared to phase lag measures (Subhani et al., 2017).

Coherence is defined as ratio of cross-spectra to the product of auto-spectra of two signals in a specific frequency band (Collura, 2008)-

$$Coherence=\;{\left|H_{uv}\right|}^{2}\;/\;\left|H_{u}\right|\left|H_{v}\right|$$

where ${|H_{uv}|}^{2}$ is the cross-spectrum between two signals, and $|H_{u}\left|,\right|H_{v}|$ are the auto-spectra of individual signals. The coherence values range between 0 and 1, where 0 indicates no linear coupling and 1 indicates maximum linear coupling. Coherence was calculated between all channels (62 × 62), for each epoch, in the six frequency bands introduced above. Significant connections were identified for each analysis using Montecarlo-based permutation statistics with the maximum correction method (max correction) implemented in Fieldtrip, and topographical plots were generated using the EEGLAB *topoplot* function.

### Data analysis

Data analysis based on ANOVAs was performed using SPSS version 29.0.0 (IBM Corp., Armonk, New York, USA). We performed Repeated Measure (RM) ANOVAs for subjective data, cortisol, and cytokines. The emotional intervention condition (with the levels control, and stress), and time (t1, t2, t3, t4, t5) served as within subject factors. RM ANOVAs were also performed for HR and HRV data with the emotional intervention condition (control, stress), and time bins (t1-t20) as within subject factors. An additional ANOVA analysis for subjective data, cortisol, HR, HRV, and cytokines was done with session order as a between-subject factor to test for session order/ carryover effects. We did not observe any significant effect of session order in our analyses (see supplementary table 4). Sphericity was tested for all ANOVAs with the Mauchly test, and Greenhouse-Geisser corrections were applied when appropriate. For post-hoc tests, Fisher’s Least Significant Difference (LSD) test was used. The critical alpha level was set at 0.05 for all tests. Correlation analyses were performed via Pearson correlations. For correlation analyses, the area under the curve (AUC) was calculated for cytokines, cortisol, HR, and HRV according to the formula (Pruessner et al., 2003) –

$$AUC_{i}=\left(\sum_{i=1}^{n-1}\frac{(m_{\left(i+1\right)}+\;\left(m_{i}\right).t_{i}}{2}\right)-\;\left(mi\;.\;\sum_{i=1}^{n-1}t_{i}\right)$$

where m_i_ denotes single measurements, t_i_ denotes the time distance between the measurements, and n denotes the total number of measurements. Correlations were then calculated based on these AUC values, similar to a previous study (Larra et al., 2023).

EEG data were analyzed and plotted using the open-source EEG data analysis software EEGLAB and Fieldtrip (Delorme & Makeig, 2004; Oostenveld et al., 2011) implemented in MATLAB (R2020b), as described above. Statistical testing for EEG power involved Monte-Carlo based permutation testing with cluster correction, implemented in the Fieldtrip toolbox in EEGLAB. Cluster-based permutation test statistics were calculated by comparing each data point resulting in a two-dimensional t-value map calculated in the electrode-frequency space, based on spatial and spectral adjacency. A cluster was then defined based on the sum of t-values from these adjacent bins. We performed 8000 random permutations to establish a distribution of cluster statistics to which each cluster t-value was compared. A threshold of alpha </= 0.01 was applied to this random distribution to identify the significant cluster. For connectivity analysis, we used a similar permutation testing method with a maximum correction method with a critical alpha level of </= 0.05 to control for familywise error rate (Delorme & Makeig, 2004; Maris & Oostenveld, 2007; Oostenveld et al., 2011)

## Author contributions

**Sumit Roy:** Conceptualization, Methodology, Software, Formal Analysis, Investigation, Writing - Original Draft, Writing - Review & Editing, Visualization **Yan Fan:** Conceptualization, Methodology, Writing - Review & Editing **Mohsen Mosayebi-Samani:** Conceptualization, Methodology, Writing - Review & Editing **Maren Claus:** Formal Analysis (Immunology), Writing - Review & Editing **Nilay Mutlu:** Investigation, Writing - Review & Editing **Thomas Kleinsorge:** Conceptualization, Methodology, Supervision, Writing - Review & Editing **Michael A. Nitsche:** Conceptualization, Methodology, Supervision, Project administration, Resources, Writing - Review & Editing

## Data availability statement

Required data can be made available upon reasonable request to the corresponding author.

## Ethics

This study was approved by the Ethics Committee of the Leibniz Research Centre for Working Environment and Human Factors at TU Dortmund (IfADo) and aligns with the Declaration of Helsinki.

## Declaration of competing interest

The authors declare the following financial interests/personal relationships which may be considered as potential competing interests: Michael A. Nitsche is a member of the Scientific Advisory Boards of Neuroelectrics, and Precisis. All other authors declare no competing interests.

## Appendix Supplementary materials

Image, application 1
