# Supplementary material for: Multimodal assessment of acute stress dynamics using an aversive video paradigm (AVP)
Source: Int J Clin Health Psychol. 2025 Jul 3;25(3):100607. doi: 10.1016/j.ijchp.2025.100607 (PMC12271860; doi:10.1016/j.ijchp.2025.100607)
Supplement: Supplementary file 1 [file mmc1.zip › Supplementary Material/Supplementary Sheet.docx]

**Supplementary Material**

**1. Movie clip details** – we have detailed the exact content of the clips

Stress clips 1 and 2 – Depiction of scenes where a female enters a subway tunnel and faces a male perpetrator who traps the female, leading to sexual abuse (which is not shown, only the leadup to that is shown); later, the male perpetrator violently attacks the female, showing intense physical violence.

Stress clips 3 and 4 – Depiction of scenes where the female victim’s boyfriend and friend go to get revenge but end up killing the wrong guy. Depiction of highly aversive male-to-male physical violence with anxiety-inducing camera angles, light, and sound effects.

Control clips 1 and 2 – Depiction of a cocktail party scene where a son meets his father after a long time, later a scene where the father wakes up at night and meets the son’s wife, who is feeling ill.

Control clips 3 and 4 – Depiction of slow scenes of the father walking around with the son’s wife and later alone to a church. Later scenes show the son going with another woman to her home.

**2. Time stamp details** – These timestamps (minute:second – minute:second) are from the commercially available DVD format of the films.

Stress clip 1 – 41:50 – 44:50

Stress clip 2 – 51:48 – 52:28; 52:47 – 53:57; 39:37 – 40:47

Stress clip 3 – 18:15 – 18:59; 19:07 – 19:20; 19:25 – 21:25

Stress clip 4 – 21:20 – 24:20

Control clip 1 – 4:16 – 7:16

Control clip 2 – 8:54 – 9:30; 32:05 – 33:25; 14:40 – 15:40

Control clip 3 – 55:40 – 58:30; 58:55 – 59:05

Control clip 4 – 29:02 – 30:11; 43:15 – 43:46; 40:36 – 41:50

Supplementary Table 1 Cluster information of the EEG power analysis during movie clip presentation. Statistics were performed between control and stress conditions for each clip individually and for all clips combined. Cluster-based permutation testing was employed to identify significant clusters in the data. This table shows information about significant clusters found in each analysis in the frequency band of interest. t-values indicate the sum of the t-values of the whole cluster, the p-value indicates the significance level, and the size of the cluster is shown by the number of the involved electrodes. The direction of the effect is indicated by the sign of the t-value, where a positive t-value indicates increased power in stress conditions and a negative t-value indicates decreased power in stress relative to control conditions. Comparisons which would include absent clusters or clusters below the threshold are not shown.

|  |  | ***All clips combined*** | ***Clip 1*** | ***Clip 2*** | ***Clip 3*** | ***Clip4*** |
| --- | --- | --- | --- | --- | --- | --- |
| **Theta (4-8 Hz)** | P value | <0.001 | 0.0034 | 0.0014 | 0.0039 | <.001 |
|  | t-value | -101.07 | -53.92 | -72.69 | -36.67 | -140.15 |
|  | No. of electrodes | 32 | 19 | 26 | 13 | 40 |
| **Alpha (8-13 Hz)** | P value | <0.001 | <0.001 | <0.001 | <0.001 | <.001 |
|  | t-value | -287.02 | -280.82 | -267.84 | -165.71 | -301 |
|  | No. of electrode | 61 | 62 | 61 | 52 | 60 |
| **Low Beta (13-15 Hz )** | P value | <0.001 | <0.001 | 0.0016 | 0.0017 | <.001 |
|  | t-value | -113.87 | -159.22 | -70.99 | -49.39 | -99.36 |
|  | No. of electrode | 34 | 47 | 21 | 18 | 31 |
| **High Beta (23-32 Hz )** | No significant clusters were observed in this frequency range | | | | | |
| **Low Gamma (32-50 Hz)** | P value | - | - | 0.002 | - | <.001 |
|  | t-value | - | - | 71.52 | - | 94.27 |
|  | No. of electrode | - | - | 26 | - | 33 |
| **High Gamma (50-80 Hz)** | P value | - | - | - | - | 0.0019 |
|  | t-value | - | - | - | - | 100.34 |
|  | No. of electrode | - | - | - | - | 33 |

Supplementary Table 2 Cluster information for EEG power analysis during resting states comparing before and after intervention. Statistics were done between RS2 and RS1 for the stress condition in EO and EC states. Cluster-based permutation testing was employed to identify significant clusters in the data. This table shows cluster information for significant clusters found in each analysis in the frequency band of interest. t-values indicate the combined t-values of the whole cluster, the p-value indicates the significance level, and the size of the cluster is shown by the number of the involved electrodes. The direction of the effect is shown by the sign of the t-value, where a positive t-value indicates increased power in the after intervention state, and a negative t-value indicates decreased power in the after intervention state relative to before intervention. Comparisons which would include absent clusters or clusters below the threshold are not shown. No clusters were observed in the control condition.

|  |  | ***Stress Eyes Closed*** | ***Stress Eyes Open*** |
| --- | --- | --- | --- |
| **Theta (4-8 Hz)** | P value | p < 0.001 | - |
|  | t-value | 202.5 | - |
|  | No. of electrode | 61 | - |
| **Alpha (8-13 Hz)** | P value | p < 0.001 | - |
|  | t-value | 242.88 | - |
|  | No. of electrode | 62 | - |
| **Low Beta (13-15 Hz)** | P value | p < 0.001 | - |
|  | t-value | 211.05 | - |
|  | No. of electrode | 61 | - |
| **High Beta (23-32 Hz)** | P value | p < 0.001 | 0.0049 |
|  | t-value | 187.38 | 91.24 |
|  | No. of electrode | 56 | 33 |
| **Low Gamma (32-50 Hz)** | P value | p < 0.001 | p < 0.001 |
|  | t-value | 209.96 | 140.86 |
|  | No. of electrode | 57 | 46 |
| **High Gamma (50-80 Hz)** | P value | p < 0.001 | p < 0.001 |
|  | t-value | 217.24 | 198.4 |
|  | No. of electrode | 59 | 58 |


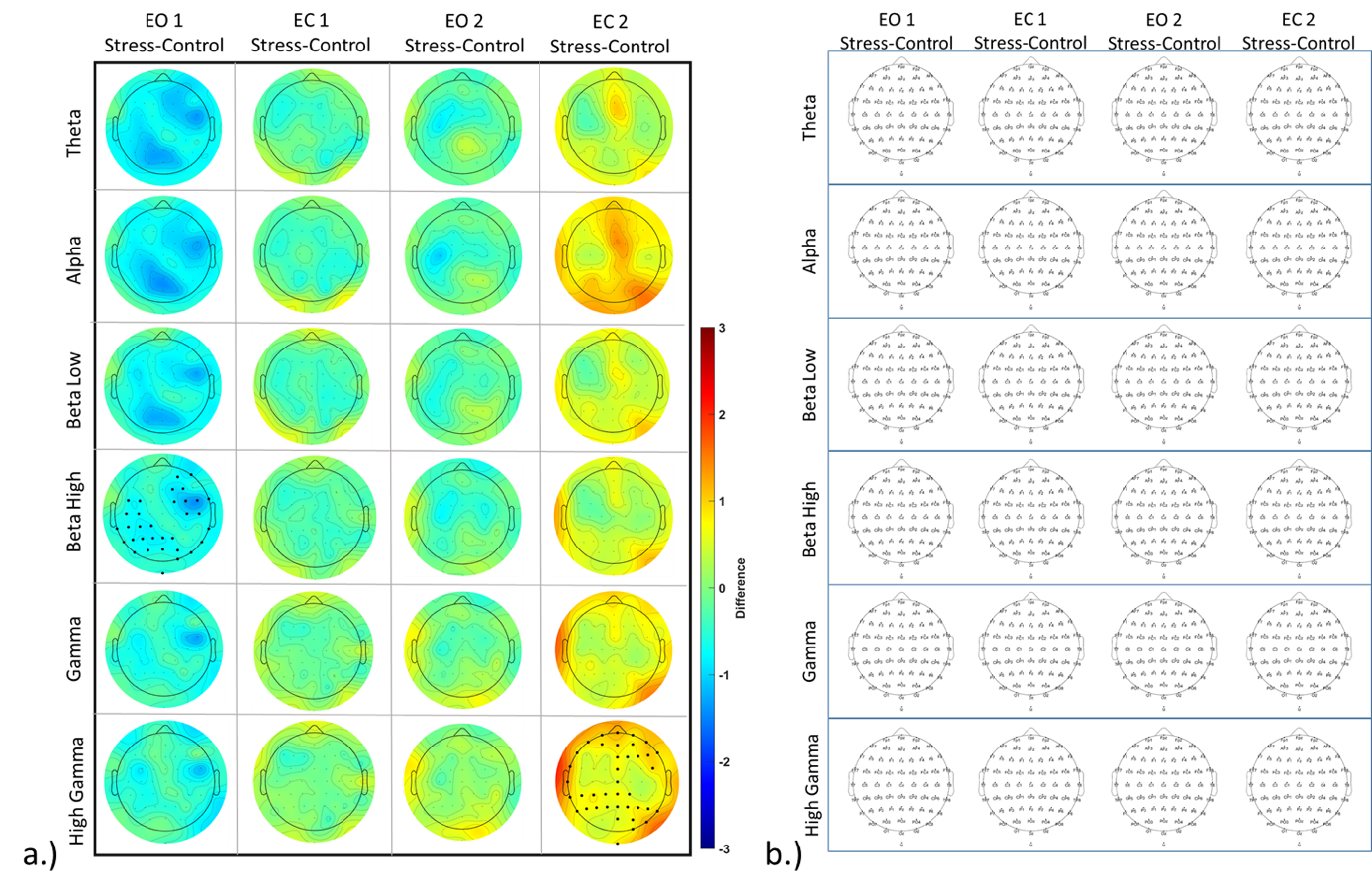


Supplementary Figure S1 a.) EEG power changes during the resting state for both, control and stress clips. The topographical plots show power differences between control and stress conditions for each resting state in EO and EC conditions. The color bar indicates the difference value range from 3 to -3. Red shows a higher value and blue a lower value in the stress compared to the control intervention condition. Statistics were done as described in clip power statistics b.) EEG connectivity difference during resting state for both, control and stress conditions. No significant connectivity change was observed. Statistics were performed as described in clip connectivity statistics. EO denotes Eyes Open and EC Eyes Closed resting conditions.

*Supplementary Table 3 Average number of epochs and channels after rejection across movie clips and RS recordings. The highest possible epochs for Clips were 90 across all clips and 60 for resting state (RS) in both Eyes Open (EO) and Eyes Closed (EC) conditions. The highest possible number of channels was 62. The mean with standard deviation (in brackets) is shown below.*

|  | **Average epochs (control condition)** | **Average epochs (stress condition)** | **Average channels (control condition)** | **Average channels (stress condition)** |
| --- | --- | --- | --- | --- |
| **Clip 1** | 88.3 (2.3) | 88.6 (1.39) | 57 (2.09) | 56.7 (2.52) |
| **Clip 2** | 88.9 (2.5) | 89 (2.17) | 57 (2.09) | 56.7 (2.52) |
| **Clip 3** | 88.3 (7.1) | 89.4 (1.43) | 57 (2.09) | 56.7 (2.52) |
| **Clip 4** | 87.16 (4.28) | 87.7 (8.78) | 57 (2.09) | 56.7 (2.52) |
| **RS1 (EO)** | 58.6 (2.37) | 58.7 (2.01) | 56.6 (3.11) | 56.3 (2.85) |
| **RS1 (EC)** | 58.9 (0.65) | 59 (1.04) | 56.2 (2,99) | 56.3 (3.1) |
| **RS2 (EO)** | 58.2 (4.36) | 58.5 (2.85) | 56.4 (3.19) | 56 (3.3) |
| **RS2 (EC)** | 59 (0.58) | 59 (0.92) | 56.1 (3.2) | 56.1 (3.51) |

*Supplementary Table 4: The main effect of session order in the additional ANOVA analyses. We did not observe any significant effect of session order on our subjective, physiological, and salivary results. d.f. = degrees of freedom, ηp^2^= partial eta squared.*

| ***The main effect of session order for -*** | ***d.f.*** | ***F-value*** | ***p-value*** | ***η^2^_p_*** |
| --- | --- | --- | --- | --- |
| *Positive Affect Scores* | *1(76)* | *.012* | *.915* | *.0* |
| *Negative Affect Scores* | *1(76)* | *1.7* | *.197* | *.02* |
| *STAI-S Anxiety Scores* | *1(76)* | *.007* | *.93* | *.0* |
| *Cortisol* | *1(68)* | *.021* | *.88* | *.0* |
| *Heart Rate* | *1(75)* | *.022* | *.882* | *.0* |
| *Heart Rate Variability* | *1(75)* | *.93* | *.33* | *.012* |
| *Cytokine – IFN-γ* | *1(70)* | *.09* | *.76* | *.001* |
| *Cytokine – IL-1β* | *1(70)* | *2.23* | *.14* | *.031* |
| *Cytokine – IL-4* | *1(70)* | *.47* | *.49* | *.007* |
| *Cytokine – IL-6* | *1(70)* | *.18* | *.67* | *.003* |
| *Cytokine – IL-8* | *1(70)* | *.95* | *.33* | *.013* |
| *Cytokine – TNFα* | *1(70)* | *.025* | *.87* | *.0* |


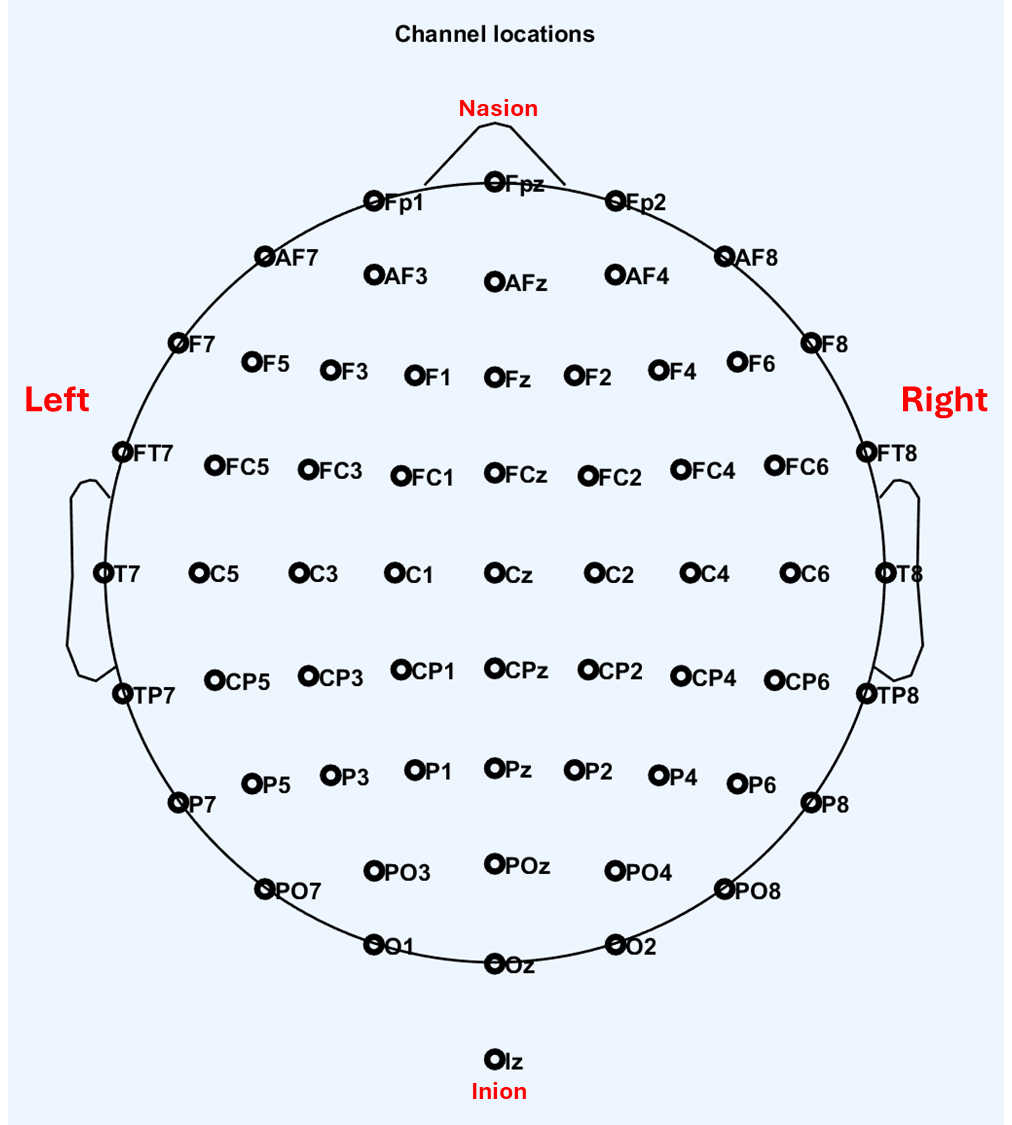


*Supplementary Figure 2 Channel locations for all EEG channels utilized in EEG power and connectivity analyses. The cartoon head is depicted from the top, with the nasion oriented upwards and the inion positioned downwards (as shown in the figure). A total of 62 electrodes were used, including 2 Electrooculography (EOG) electrodes (one vertical and one horizontal EOG, channels not shown), one ground electrode over the left cheek, and one reference electrode placed over the left mastoid. Electrode locations are shown by a black circle, with the electrode name shown next to it. Positions of the left and right hemispheres are shown.*
